# Metallothioneins Are Required for Formation of Cross-Adaptation Response to Neurobehavioral Toxicity from Lead and Mercury Exposure in Nematodes

**DOI:** 10.1371/journal.pone.0014052

**Published:** 2010-11-18

**Authors:** Boping Ye, Qi Rui, Qiuli Wu, Dayong Wang

**Affiliations:** 1 College of Life Science and Technology, China Pharmaceutical University, Nanjing, China; 2 Key Laboratory of Developmental Genes and Human Disease in Ministry of Education, Institute of Life Sciences, Southeast University, Nanjing, China; 3 College of Life Sciences, Nanjing Agricultural University, Nanjing, China; East Carolina University, United States of America

## Abstract

Metallothioneins (MTs) are small, cysteine-rich polypeptides, but the role of MTs in inducing the formation of adaptive response is still largely unknown. We investigated the roles of metallothionein genes (*mtl-1* and *mtl-2*) in the formation of cross-adaptation response to neurobehavioral toxicity from metal exposure in *Caenorhabditis elegans*. Pre-treatment with mild heat-shock at L2-larva stage effectively prevented the formation of the neurobehavioral defects and the activation of severe stress response in metal exposed nematodes at concentrations of 50 and 100 µM, but pre-treatment with mild heat-shock did not prevent the formation of neurobehavioral defects in 200 µM of metal exposed nematodes. During the formation of cross-adaptation response, the induction of *mtl-1* and *mtl-2* promoter activity and subsequent GFP gene expression were sharply increased in 50 µM or 100 µM of metal exposed P*mtl-1*::GFP and P*mtl-2*::GFP transgenic adult animals after mild heat-shock treatment compared with those treated with mild heat-shock or metal exposure alone. Moreover, after pre-treatment with mild heat-shock, no noticeable increase of locomotion behaviors could be observed in metal exposed *mtl-1* or *mtl-2* mutant nematodes compared to those without mild heat-shock pre-treatment. The defects of adaptive response to neurobehavioral toxicity induced by metal exposure formed in *mtl-1* and *mtl-2* mutants could be completely rescued by the expression of *mtl-1* and *mtl-2* with the aid of their native promoters. Furthermore, over-expression of MTL-1 and MTL-2 at the L2-larval stage significantly suppressed the toxicity on locomotion behaviors from metal exposure at all examined concentrations. Therefore, the normal formation of cross-adaptation response to neurobehavioral toxicity induced by metal exposure may need the enough accumulation of MTs protein in animal tissues.

## Introduction

An adaptive response is a phenomenon in which a sub-lethal or non-lethal pre-treatment causes an increased resistance when an organism is challenged with higher doses or concentrations of that particular agent [1]. Such an adaptive response to oxidative damage occurs in a variety of organisms [2]–[4]. In addition to conferring protection against the same agent, cross adaptation can usually occur. Cross-adaptation response is defined as the capacity of cells or organisms to become resistant to a lethal agent when pretreated with a different lethal substance [1]. In particular, since organisms live in an environment where the threat of oxidative damage is continual, cellular and molecular mechanisms may have evolved to avoid and repair this damage and pre-exposure to mild stress may confer resistance to agents [5]–[6].

In nematode *Caenorhabditis elegans*, non-lethal stress such as mild heat shock can also have beneficial effects on stress resistance and longevity [7]–[8]. Lithgow et al. (1995) investigated the relationship between increased thermotolerance and life-span by developing conditions for environmental induction of thermotolerance, and found that the pretreatment at sub-lethal temperature induces increased thermotolerance and small but statistically highly significant increase in life expectancy [9]. Pre-exposure of wild-type nematodes to oxygen can confer a protective effect against the lethality imposed by subsequent X-irradiation [1]. Short-term exposure to hyperoxia can further extend the life span of *age-1*, and *age-1* mutant also show resistance to paraquat and heat shock [10]. Moreover, young nematodes adapted the oxidative stress induced by the quinine plumbagin or hyperoxia treatment by increasing their content of superoxide dismutase (SOD) and they survived; whereas older nematodes did not induce SOD and suffered loss of viability, suggesting the adaptation to oxidative stress in young, but not in mature or old *C. elegans* [11]. Furthermore, pre-treatment with mild UV irradiation suppresses reproductive toxicity induced by subsequent cadmium in nematodes [12].

*C. elegans*, a free-living soil nematode, is an excellent model organism because of its short lifespan, ease of manipulation, and low cost. It has been found favor as a valuable bioindicator organism in toxicological study and ecological assessment for its best-characterized properties at the genetic, physiological, molecular, and developmental levels [13]–[14]. So far, the heavy metal toxicity and contamination can be effectively detected by the endpoints of mortality [15]–[16], lifespan [17]–[19], reproduction [20]–[21], and feeding [21]–[22] in *C. elegans*. Because the nematode behaviors can be easily monitored under the microscope, another sensitive endpoint, movement or locomotion behavior, was also monitored using a computer tracking system after metal exposure in nematodes [23]–[26]. In addition, Wang and Xing (2008) have examined the endpoints of head thrash, body bend, and basic movements in metal exposed nematodes, and indicated that the endpoints of head thrash, body bend, and forward turn can establish a fast and economic way to assess the presence of acute toxicity from heavy metal exposure [27]. More recently, it was reported that pre-treatment with mild metal exposure can activate the adaptive response to neurotoxicity of locomotion behavior induced by subsequent severe metal exposure in nematodes [28]. Moreover, pre-treatment with mild UV irradiation increases the resistance of nematodes to toxicity on locomotion behaviors from metal exposure [29].

Metallothioneins (MTs) are small (∼60 amino acid residues), cysteine-rich (∼30%) polypeptides that avidly bind 7–12 M of transition metal/M of protein via thiolate bonds, and exist in a wide range of organisms, including higher and lower eukaryotes, and even some prokaryotes [30]–[31]. MTs may be involved in the detoxication of heavy metals, such as cadmium (Cd) and mercury (Hg), the homoeostasis of essential metals such as zinc (Zn) and copper (Cu), and the protection against intracellular oxidative damage [32]–[33]. The MT transcription can be induced by metal exposure, ionizing radiation, heat-shock, and oxidative stress [33]–[35]. In *C. elegans*, two MT genes, designated *mtl-1* and *mtl-2*, have been identified and characterized [32]. However, it is still largely unknown whether the MTs can regulate the formation of adaptive response in *C. elegans*.

Thus, in this study, we first investigated whether pre-treatment of heat shock can confer a protective effect against the neurobehavioral toxicity induced by subsequent metal exposure in nematodes. Moreover, we examined the possible important roles of *C. elegans* MTs in regulating the formation of cross-adaptation response to the neurobehavioral toxicity induced by subsequent metal exposure. Our data suggest that MTs are essential for the formation of cross-adaptation response to neurobehavioral toxicity induced by metal exposure in *C. elegans*.

## Results

### Locomotion behavior activities in heat-shock treated wild-type N2 nematodes

**Figure 1:**
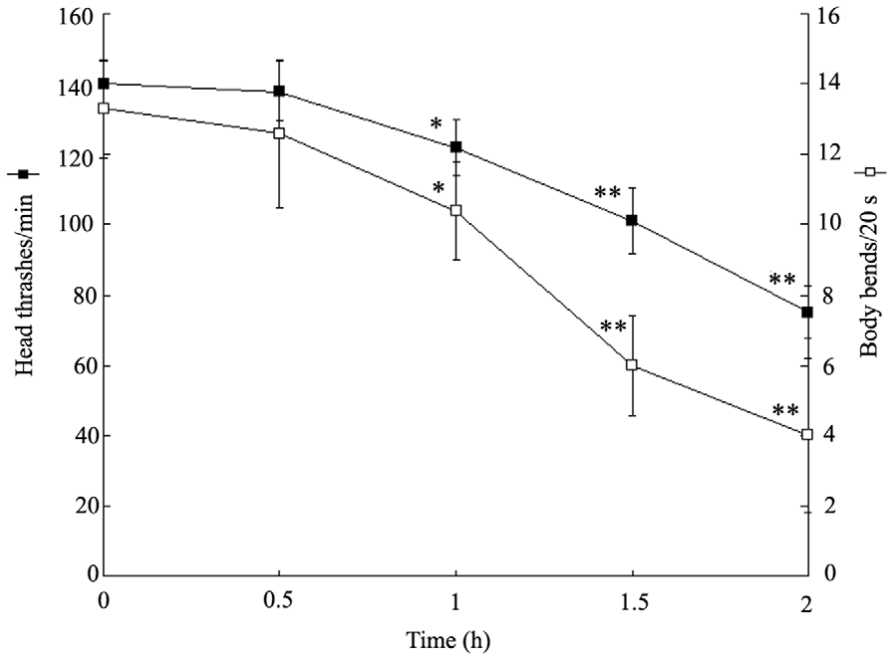
Neurobehavioral toxicity induced by heat-shock in wild-type N2 nematodes. L4-stage larvae animals were heat stressed for 0.5, 1, 1.5, 2 h at 36°C. Bars represent means ± S.D. * *p*<0.05 *vs* 0 h; ** *p*<0.01 *vs* 0 h.

In the present study, the locomotion behavior activities were assessed by the endpoints of head thrash and body bend. As shown in Fig. 1, in wild-type nematodes, no obvious alterations of head thrash were recorded in nematodes treated with heat-shock for 0.5 h. The most significant (*p*<0.01) decreases of head thrashes were observed in heat-shock treated nematodes for 1.5 and 2 h at 36°C compared to control. Similarly, no remarkable changes of body bend were formed in nematodes treated with heat-shock for 0.5 h, and the very noticeable (*p*<0.01) reduction of body bends was also found in heat-shock treated nematodes for 1.5 and 2 h compared to control. Especially, only moderate, but significant (*p*<0.05) reduction of head thrash or body bend was observed in heat-shock treated nematodes for 1 h compared to control. Therefore, 1-h of heat-shock treatment will induce the mild reduction of locomotion behaviors in nematodes.

### The alterations of stress response in heat-shock treated wild-type N2 nematodes

**Figure 2:**
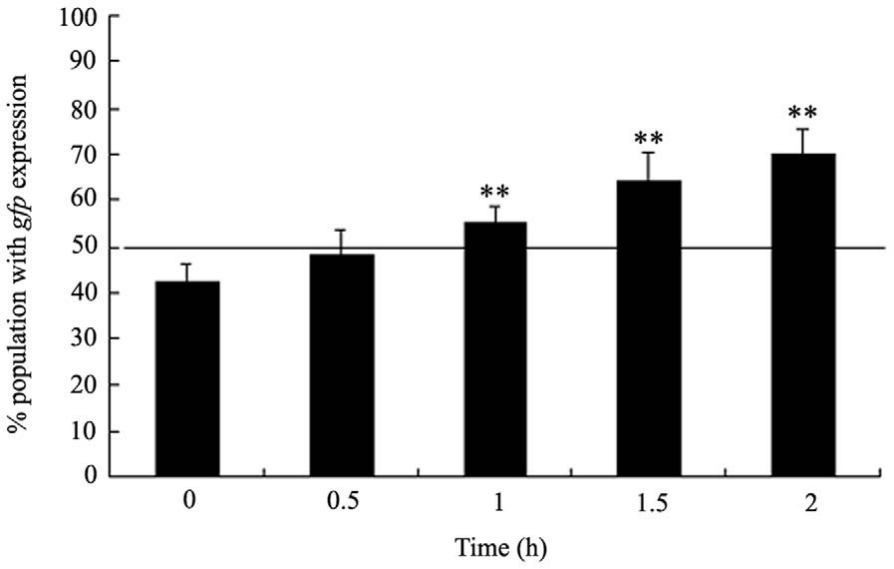
Stress response in heat-shock treated wild-type N2 nematodes. L4-stage larvae animals were heat stressed for 0.5, 1, 1.5, 2 h at 36°C. To evaluate the stress response, significant induction of *hsp-16.2*::*gfp* expression (50% of a population, above the line) was observed in wild-type N2 animals. Bars represent means ± S.D. * *p*<0.05 *vs* 0 h; ** *p*<0.01 *vs* 0 h.

It was reasoned that if the stress exposure was toxic, it would thus result in a stress response as detected by the expression of HSP-16 expression [16]. Again, we explored one of the stable transgenic lines of *hsp-16.2::gfp* to investigate the stress response induced by heat-shock. As shown in Fig. 2, exposure to heat-shock for 0.5 h would not result in a significant induction of *hsp-16.2*::*gfp* expression (50% of a population, above the line), whereas treatment with both heat-shock for 1.5 and 2 h caused a sharp (*p*<0.01) elevation of *hsp-16.2*::*gfp* expression compared to control. Moreover, treatment with 1-h of heat-shock caused a moderate, but significant (*p*<0.01) induction of *hsp-16.2*::*gfp* expression compared to control. Thus, 1-h of heat-shock treatment will result in the mild stress response in nematodes.

### Pre-treatment with mild heat-shock activates the adaptive response to neurobehavioral toxicity induced by metal exposure in wild-type N2 nematodes

**Figure 3:**
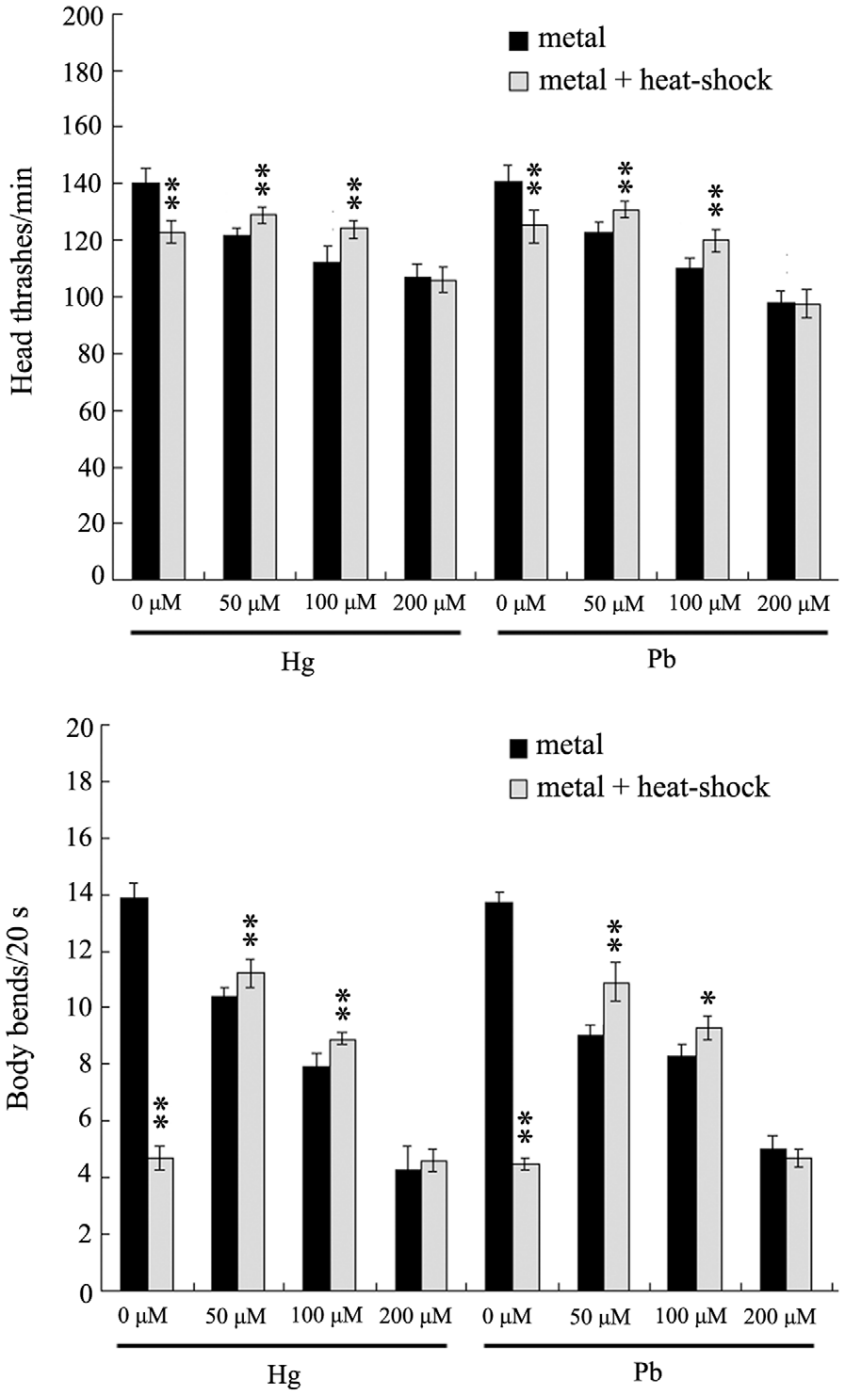
Effects of pre-treatment with mild heat-shock on neurobehavioral toxicity induced by metal exposure in wild-type N2 nematodes. L2-stage larvae animals were heat stressed for 1 h at 36°C. The exposed metal concentrations were 50, 100, and 200 µM. Bars represent means ± S.D. * *p*<0.05 *vs* metal; ** *p*<0.01 *vs* metal.

Previous studies have demonstrated that metal exposure would cause severe neurobehavioral toxicity in nematodes [22]–[26]. As shown in Fig. 3, in wild-type nematodes, exposure to metals of Hg and Pb at concentrations of 50, 100, and 200 µM will noticeably suppress the head thrashes and body bends compared to control. Moreover, pre-treatment with heat-shock for 1 h at L2-larva stage significantly (*p*<0.01) suppressed the decreases of head thrash and body bend formed in metal (Hg and Pb) exposed nematodes at the concentration of 50 µM. Similarly, pre-treatment with heat-shock for 1 h also markedly (*p*<0.01) inhibited the reductions of head thrash and body bend induced by metal exposure at the concentration of 100 µM. In contrast, pre-treatment with heat-shock for 1 h did not obviously influenced the occurrence of neurobehavioral defects formed in metal (Hg and Pb) exposed nematodes at the concentration of 200 µM compared to those without heat-shock pre-treatment. These data imply that pre-treatment with mild heat-shock can largely suppress the formation of neurobehavioral defects induced by metal exposure at the concentrations of 50 µM and 100 µM. We selected the concentrations of 50 µM and 100 µM to perform the following experiments on the adaptive response.

### Effects of pre-treatment with mild heat-shock on stress responses induced by metal exposure in wild-type N2 nematodes

**Figure 4:**
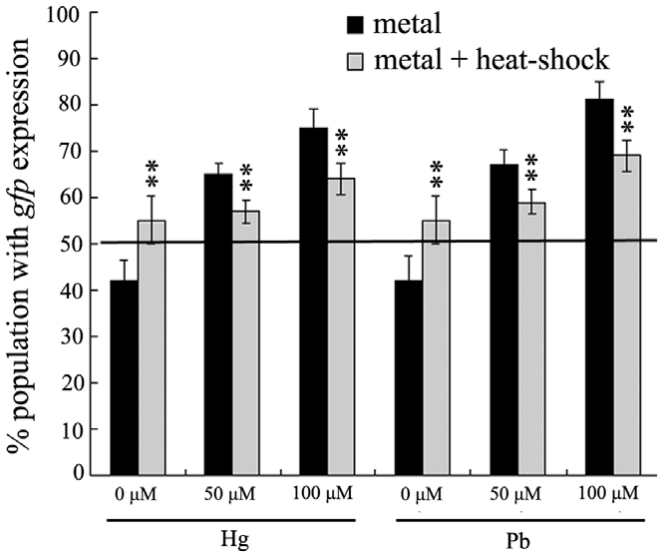
Effects of pre-treatment with mild heat-shock on the stress responses induced by metal exposure in wild-type N2 nematodes. L2-stage larvae animals were heat stressed for 1 h at 36°C. The exposed metal concentrations were 50 and 100 µM. To evaluate the stress response, significant induction of *hsp-16.2*::*gfp* expression (50% of a population, above the line) was observed in wild-type N2 animals. Bars represent means ± S.D. * *p*<0.05 *vs* metal; ** *p*<0.01 *vs* metal.

To examine the role of stress response in inducing the adaptation to metal toxicity after pre-treatment with mild heat-shock, we next investigated the effects of pre-treatment with mild heat-shock on the expression of *hsp-16.2::gfp* in metal exposed nematodes. *hsp-16.2* is ubiquitously expressed throughout most somatic tissues, and the induced *hsp-16.2::gfp* expression signal at the pharyngeal bulb was strong and allowed a distinct identification of induced expression by stresses [16]. Previous study has indicated that a low level of background *gfp* expression in about 40% of the *hsp-16.2::gfp* transgenic nematodes was observed, although all the transgenic nematodes responded positively after metal exposure [16]. To distinguish the positive results from the background in the stress test, only stresses that could induce 50% of the transgenic nematodes to display strong *hsp-16.2::gfp* expression signal at the pharyngeal bulb were taken as having positive effect [16]. As shown in Fig. 4, metal (Hg and Pb) exposure at the concentrations of 50 µM and 100 µM induced the noticeable elevation of *hsp-16.2::gfp* expression. Furthermore, pre-treatment with heat-shock for 1 h at the L2-larva stage significantly reduced the percentage of population with *hsp-16.2::gfp* expression in examined metal exposed nematodes at the concentrations of 50 µM (*p*<0.01) and 100 µM (*p*<0.01) compared to those without heat-shock pre-treatment; however, no obvious alteration of the percentage of population with *hsp-16.2::gfp* expression was observed in metal exposed nematodes at the concentration of 200 µM after mild heat-shock pre-treatment (data not shown). Therefore, pre-treatment with mild heat-shock will largely suppress the metal-induced stress response in nematodes.

#### Alterations of MTs gene expression during the formation of cross-adaptation response to neurobehavioral toxicity induced by metal exposure

**Figure 5:**
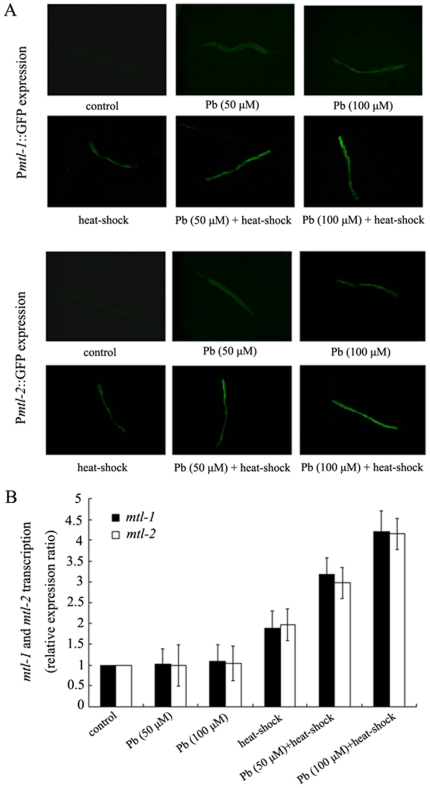
Induction of metallothionein gene expression during the formation of cross-adaptation response to neurobehavioral toxicity induced by Pb exposure. (A) Induction of green fluorescence protein in the P*mtl-1::GFP* and P*mtl-2::GFP* transgenic nematodes. (B) *mtl-1* and *mtl-2* transcription assay. Relative expression ratio (between *mtl-1* or *mtl-2* gene and *ubq-1* reference gene) in treatments are normalized to the control. L2-stage larvae animals were heat stressed for 1 h at 36°C. The exposed metal concentrations were 50 and 100 µM. Bars represent means ± S.D.

MTs are considered to be involved in the detoxification of and protection from some heavy metals, such as cadmium and mercury [31]–[32], [34]–[36]. The nematode *C. elegans* has two MTs, *mtl-1* and *mtl-2*, with the similar functions [31], [34]–[35]. Moreover, it has been implied that the adaptation to elevated environmental metal concentrations may be achieved through the increased synthesis of MT or via the duplication of MT genes [37]–[38]. We further examined the possible alterations of MTs gene expression during the formation of cross-adaptation response to neurobehavioral toxicity induced by metal exposure. The two nematode strains, P*mtl-1*::GFP and P*mtl-2*::GFP, were constructed to have GFP protein fused to the promoters of *mtl-1* and *mtl-2*, respectively. As shown in Fig. 5A, the GFP signal was nearly undetectable in control animals. The mild heat-shock treatment induced the moderate but significant increase of GFP signals in P*mtl-1*::GFP and P*mtl-2*::GFP transgenic strains. Exposure to 50 µM and 100 µM of Pb induced the slight increase of GFP signals in P*mtl-1*::GFP and P*mtl-2*::GFP transgenic strains. In contrast to these, after mild heat-shock treatment at the L2-larval stage, the GFP signals were sharply increased in 50 µM or 100 µM of Pb exposed P*mtl-1*::GFP and P*mtl-2*::GFP transgenic adult animals compared with those treated with mild heat-shock or Pb exposure alone. We have obtained 5 lines of P*mtl-1*::GFP and 7 lines of P*mtl-2*::GFP transgenic nematodes, and all showed the similar phenotypes (data not shown). The similar observations were obtained in Hg exposed nematodes after mild heat-shock pre-treatment (data not shown). These data imply that the formation of cross-adaptation response to neurobehavioral toxicity induced by metal exposure may be closely associated with the induction of *mtl-1* and *mtl-2* promoter activity and subsequent GFP gene expression. Similarly, as shown in Fig. 5B, exposure to 50 µM and 100 µM of Pb did not significantly increase the expression of *mtl-1* and *mtl-2* at the transcription level; however, after mild heat-shock treatment at the L2-larval stage, the *mtl-1* or *mtl-2* expression at the transcription level was obviously increased in 50 µM or 100 µM of Pb exposed nematodes compared with those treated with mild heat-shock or Pb exposure alone. We next selected the Pb to further investigate the roles of MTs in regulating the formation of cross-adaptation response to neurobehavioral toxicity induced by metal exposure in *C. elegans*.

### Effects of pre-treatment with mild heat-shock on neurobehavioral toxicity induced by Pb exposure in *mtl-1* and *mtl-2* mutant nematodes

**Figure 6:**
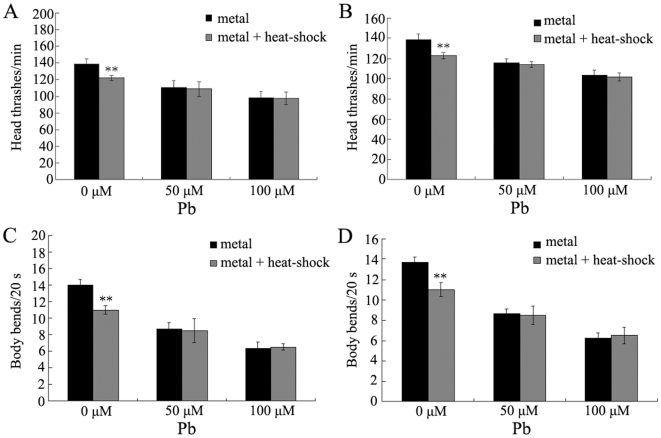
Effects of pre-treatment with mild heat-shock on neurobehavioral toxicity in Pb exposed *mtl-1(tm1770)* and *mtl-2(gk125)* mutant nematodes. (A) Effects of pre-treatment with mild heat-shock on head thrash in Pb exposed *mtl-1(tm1770)* mutant nematodes. (B) Effects of pre-treatment with mild heat-shock on head thrash in Pb exposed *mtl-2(gk125)* mutant nematodes. (C) Effects of pre-treatment with mild heat-shock on body bend in Pb exposed *mtl-1(tm1770)* mutant nematodes. (D) Effects of pre-treatment with mild heat-shock on body bend in Pb exposed *mtl-2(gk125)* mutant nematodes. L2-stage larvae animals were heat stressed for 1 h at 36°C. The exposed metal concentrations were 50 and 100 µM. Bars represent means ± S.D. ** *p*<0.01 *vs* metal.

We further investigated the possible alterations of neurobehavioral toxicity in Pb (50 µM and 100 µM) exposed *mtl-1* and *mtl-2* mutants after pre-treatment with mild heat-shock. As shown in Fig. 6, mutations of *mtl-1* and *mtl-2* did not obviously affect the nematode locomotion behaviors compared to wild-type, and treatment with heat-shock for 1 h at the L2-larva stage caused a moderate but significant (*p*<0.01) decrease of head thrashes and body bends in *mtl-1(tm1770)* and *mtl-2(gk125)* mutant nematodes compared with those observed in wild-type nematodes. Moreover, after pre-treatment with mild heat-shock, no noticeable increase of head thrashes and body bends were detected in Pb exposed *mtl-1(tm1770)* and *mtl-2(gk125)* mutant nematodes compared with those without mild heat-shock pre-treatment. Therefore, mutations of *mtl-1* and *mtl-2* genes result in the alterations of cross-adaptation response to neurobehavioral toxicity induced by Pb exposure in nematodes. In addition, we did not observed the obvious increase of head thrashes and body bends in 200 µM of Pb exposed *mtl-1(tm1770)* and *mtl-2(gk125)* mutant nematodes after pre-treatment with mild heat-shock compared with those without mild heat-shock pre-treatment (data not shown).

### Rescue assay on the deficits in adaptive response to neurobehavioral toxicity induced by Pb exposure formed in *mtl-1* and *mtl-2* mutant nematodes

**Figure 7:**
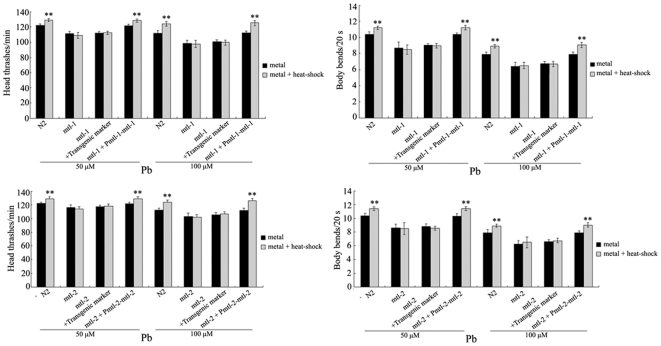
The defects of adaptive response to neurobehavioral toxicity induced by Pb exposure in *mtl-1(tm1770)* and *mtl-2(gk125)* mutants can be rescued by the expression of *mtl-1* and *mtl-2* with their native promoters. L2-stage larvae animals were heat stressed for 1 h at 36°C. The exposed metal concentrations were 50 and 100 µM. The plasmids were injected as a mix at 20 ng/µl using P*dop-1::rfp* as a transgenic marker. Bars represent means ± S.D. ** *p*<0.01 *vs* metal.

To confirm the important roles of *mtl-1* and *mtl-2* genes in regulating the adaptive response to neurobehavioral toxicity observed in *mtl-1(tm1770)* and *mtl-2(gk125)* mutants, we next performed the rescue experiments in nematodes. As shown in Fig. 7, transgene only with the vector of P*dop-1::rfp* (P*dop-1*, *dop-1* promoter), which served as a transgenic marker, did not affect the formation of adaptive response to neurobehavioral toxicity induced by metal expression. The defects of adaptive response to neurobehavioral toxicity on head thrash or body bend induced by Pb exposure at the concentrations of 50 µM and 100 µM formed in *mtl-1(tm1770)* mutant were completely rescued by the expression of *mtl-1* with the aid of its native promoter. Similarly, the defects of adaptive response to neurobehavioral toxicity on head thrash or body bend induced by Pb exposure at the concentrations of 50 µM and 100 µM formed in *mtl-2(gk125)* mutant were also completely rescued by the expression of *mtl-2* with the aid of its native promoter. We have obtained 4 lines of P*mtl-1-mtl-1* and 6 lines of P*mtl-2-mtl-2* transgenic nematodes, and all showed the similar phenotypes (data not shown).

**Figure 8:**
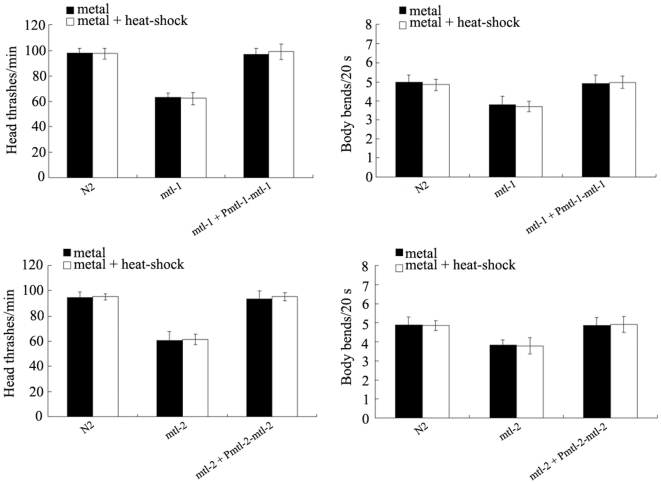
The defects of adaptive response to neurobehavioral toxicity induced by exposure to 200 µM of Pb in *mtl-1(tm1770)* and *mtl-2(gk125)* mutants can not be rescued by the expression of *mtl-1* and *mtl-2* with their native promoters. L2-stage larvae animals were heat stressed for 1 h at 36°C. The plasmids were injected as a mix at 20 ng/µl using P*dop-1::rfp* as a transgenic marker. Bars represent means ± S.D.

Interestingly, after mild heat-shock treatment at the L2-larval stage, we further observed the different phenotypes of locomotion behavior in 200 µM of Pb exposed *mtl-1* and *mtl-2* adult mutants. As shown in Fig. 8, the defects of adaptive response to neurobehavioral toxicity on head thrash or body bend induced by Pb exposure at the concentration of 200 µM formed in *mtl-1(tm1770)* mutant could not be rescued by the expression of *mtl-1* with the aid of its native promoter. Similarly, the defects of adaptive response to neurobehavioral toxicity on head thrash or body bend induced by Pb exposure at the concentration of 200 µM formed in *mtl-2(gk125)* mutant could not also be rescued by the expression of *mtl-2* with the aid of its native promoter. Therefore, the expression of *mtl-1* and *mtl-2* with their native promoters can not rescue the defects of adaptive response to neurobehavioral toxicity induced by exposure to 200 µM of Pb in *mtl-1* and *mtl-2* mutants.

### Over-expression of MTL-1 or MTL-2 at the L2-larval stage suppresses the neurobehavioral toxicity induced by Pb exposure

**Figure 9:**
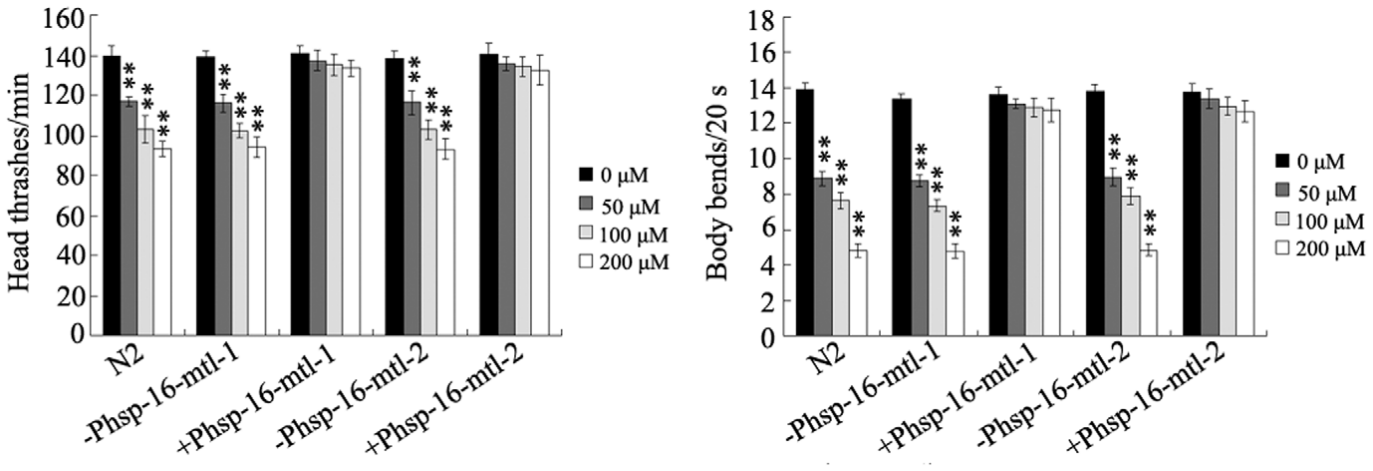
Effects of MTL-1 and MTL-2 over-expression at the L2-larval stage on the neurobehavioral toxicity induced by Pb exposure. –P*hsp-16-mtl-1* and –P*hsp-16-mtl-2*, wild-type animals carrying P*hsp-16-mtl-1* or –P*hsp-16-mtl-2* without heat-shock treatment; –P*hsp-16-mtl-1* and –P*hsp-16-mtl-2*, wild-type animals carrying P*hsp-16-mtl-1* or –P*hsp-16-mtl-2* treated with heat-shock (30°C, 4-hr) at the L2-larval stage. Bars represent means ± S.D. ** *p*<0.01 *vs* 0 µM.

We finally investigated the effects of MTL-1 and MTL-2 over-expression on the neurobehavioral toxicity from Pb exposure. To induce the over-expression of MTL-1 and MTL-2 at the L2-larval stage, MTL-1 and MTL-2 were expressed in the wild-type N2 animals using a heat shock promoter. The transgenic animals were treated by heat-shock at the L2-larval stage, and the treated animals were further exposed to metal solutions at the L4-larval stage. Under our experimental conditions, the wild-type nematodes treated with heat-shock at 30°C for 4-hr showed normal locomotion behaviors (data not shown). As shown in Fig. 9, transgenes with P*hsp-mtl-1* and P*hsp-mtl-2* did not obviously influence the locomotion behaviors (head thrash and body bend) of Pb exposed nematodes without heat-shock treatment. In contrast, over-expression of MTL-1 and MTL-2 by heat-shock treatment at the L2-larval stage significantly suppressed the toxicity on locomotion behaviors from Pb exposure at all examined concentrations. More interestingly, the over-expression of MTL-1 and MTL-2 by heat-shock treatment at the L2-larval stage even noticeably inhibited the toxicity on locomotion behaviors from Pb exposure at the concentration of 200 µM.

## Discussion

The neurotoxicity from metal exposure on locomotion behaviors has been widely studied for many years. In this project, we selected the metals of Hg and Pb to study the adaptation response of nematodes to neurobehavioral toxicity induced by metal exposure after mild stress pre-treatment. These metals are ubiquitous toxicants that will result in a wide range of adverse health effects in humans, and inorganic metals from natural and man-made sources are released into air, soil and water. The severe deficits in locomotion behaviors have been observed in Hg and Pb exposed *C. elegans* [27], [39]. In this study, we provide evidence to raise such a notion that the neurobehavioral toxicity induced by metal exposure can be effectively prevented by pre-treatment with mild heat-shock, which provides the nematodes an important strategy to resist the neurotoxicity from metal exposure or other stresses. This conclusion is consistent with our previous observation that pre-treatment with mild UV irradiation increases the resistance of nematodes to toxicity on locomotion behaviors from metal exposure [29].

In the present study, we examined the possible roles of mild heat-shock in inducing the adaptive response to neurobehavioral toxicity from metal exposure. The locomotion behavior was assessed by the endpoints of head thrash and body bend, which have been successfully explored to evaluate the neurobehavioral toxicity induced by metal exposure [17]-[18]. The heat-shock is a commonly and extensively investigated stress in nematodes [9]. Previous studies usually explored the heat-shock for 2 h as a severe stress [9]. Our data suggest that treatment with heat-shock for 2 h could further induce severe deficits in locomotion behaviors, whereas treatment with heat-shock for 1 h could only cause mild toxicity on locomotion behaviors in exposed nematodes. Thus, we here selected the heat-shock for 1 h for the pre-treatment to study the adaptive response of nematode to neurobehavioral toxicity from metal exposure. Moreover, our previous studies suggest that exposure to more than 50 µM of metals often resulted in severe defects of locomotion behaviors; however, exposure to 2.5 µM of metals would induce moderate but significant decrease of locomotion behaviors (27). Therefore, the concentrations of 50, 100, and 200 µM were selected further to evaluate the neurobehavioral toxicity induced by metal exposure. In addition, all examinations were performed at larval stages (L2 or L4), since Darr and Fridovich (1995) indicated that the adaptation to oxidative stress exists in young, but not mature or old *C. elegans* [11]. Our previous study further suggested that exposure to Pb and Hg in L2-larvae can induce more severe deficits in locomotion behaviors than adult nematodes [23].

Our data suggest that pre-treatment with mild heat-shock will largely increase the resistance of nematodes to neurobehavioral toxicity induced by metal exposure, and suppress the formation of metal-induced stress response in nematodes, suggesting that pre-treatment with mild stresses can activate the adaptive response of nematodes to neurobehavioral toxicity from metal exposure by suppressing the severe stress response formed in metal exposed nematodes. Together with our previous observations [28]–[29], both the cross adaptive response and the non-cross adaptive response to neurobehavioral toxicity induced by metal exposure can be formed in nematodes. Thus, the induction of adaptive response to severe forms of exposure by suppressing the stress response should be a conserved mechanism after pre-treatment with mild stress. Furthermore, our data demonstrate that pre-treatment with severe stress can not effectively induce the adaptive response to neurobehavioral toxicity from metal exposure, and the adaptive response can also not be effectively activated by mild stress pre-treatment in nematodes with a mostly severe impairment from metal exposure. Especially, pre-treatment with severe heat-shock resulted in significant decrease of locomotion behavior (head thrashe and body bend) in metal exposed nematodes at different concentrations (D-Y Wang et al., personal communication), implying that the adaptive response might be activated but was not effective enough in protecting against the heavy metal toxicity. Thus, the suitable and mildly toxic pre-exposure is essential for the activation of adaptive response to neurobehavioral toxicity from metal exposure in nematodes.

Furthermore, our data suggest that MTs are essential for the formation of cross-adaptation response to neurobehavioral toxicity induced by metal exposure in *C. elegans*. MT proteins play a major role in metal detoxification in nematodes [34]–[36], [40]. It was also reported that the MT-null mice seemed more susceptible to Hg^0^-induced neurobehavioral toxicity than the wild-type, and the increased susceptibility to MT-null females to behavioral changes caused by prenatal Hg^0^ exposure is due to a greater retention of mercury [41]–[42]. The adaptive response to the neurobehavioral toxicity induced by metal exposure can not be detected in *mtl-1* and *mtl-2* mutant nematodes. The deficits in the formation of cross-adaptation response to neurobehavioral toxicity induced by exposure to examined metals at concentrations of 50 and 100 µM in *mtl-1* and *mtl-2* mutants could be further rescued by the expression of MTL-1 and MTL-2 with the aid of their own native promoters. These data imply that the adaptive response to neurobehavioral toxicity induced by metal exposure could not be effectively activated in *mtl-1* and *mtl-2* mutant nematodes. Similarly, the adaptive response to X-radiation exists in wild-type nematode that is also not present in *rad-1* and *rad-2* mutants with the properties of UV and ionizing radiation hypersensitive [1].

*C. elegans* MT1 is constitutively expressed in three cells of the posterior bulb of the pharynx but is also induced by metal exposure and heat-shock in intestinal cells, and MT2 expression occurs only in intestinal cells in metal exposed nematodes [32]–[34]. In addition, significant levels of intestinal cell transcription of *C. elegans* MTs are difficult to be observed in the absence of stress [32]–[34]. In the present study, our data demonstrate that the defects of adaptive response to neurobehavioral toxicity induced by metal exposure formed in *mtl-1* and *mtl-2* mutants could be not only completely rescued by the expression of *mtl-1* or *mtl-2* with the aid of its own promoter, but also largely rescued by the expression of *mtl-1* or *mtl-2* gene in the nervous system (D.-Y. Wang et al., personal communication). In contrast, expression of MTL-1 and MTL-2 in the muscle cells could not rescue the deficits in the formation of cross-adaptation response to neurobehavioral toxicity induced by metal exposure in the *mtl-1* and *mtl-2* mutant nematodes (D.-Y. Wang et al., personal communication). These data imply that *mtl-1* and *mtl-2* genes may function cell non-autonomously in regulating the formation of cross-adaptation response to neurobehavioral toxicity induced by metal exposure in nematodes.

During the formation of cross-adaptation response to neurobehavioral toxicity induced by metal exposure, we further observed that the induction of *mtl-1* and *mtl-2* promoter activity and subsequent GFP gene expression were sharply increased in 50 µM or 100 µM of metal exposed P*mtl-1*::GFP and P*mtl-2*::GFP transgenic adult animals after mild heat-shock treatment at the L2-larval stage compared with those treated with mild heat-shock or metal exposure alone. Previous study from Ma et al. (2009) further supported our observations on the expression of P*mtl-1*::GFP and P*mtl-2*::GFP in Pb and Hg exposed nematodes, and help to explain our data presented in this study [43]. These data strongly imply that the normal formation of cross-adaptation response to neurobehavioral toxicity induced by metal exposure may require the enough accumulation of MTs protein in animal tissues. Nevertheless, expression of MTL-1 and MTL-2 did not rescue the defects of adaptive response to neurobehavioral toxicity induced by exposure to 200 µM of Pb in *mtl-1* and *mtl-2* mutants. Moreover, we observed that over-expression of MTL-1 and MTL-2 at the L2-larval stage significantly suppressed the toxicity on locomotion behaviors from Pb exposure at all examined concentrations. In contrast, expression of proteins not related with stress response and adaptive response, such as MOD-5, a serotonin transporter, and EAT-4, a glutamine transporter, at the L2-larval stage did not influence the toxicity on locomotion behaviors from Pb exposure at all examined concentrations (D.-Y. Wang et al., personal communication). Therefore, pre-treatment with mild stress may also activate some unknown mechanism, which will further enhance the MTs accumulation while the animals are further exposure to very severe stresses.

In conclusion, pretreatment with mild heat-shock will activate the resistance of nematodes to neurobehavioral toxicity from metal exposure, and MT genes are required for the formation of cross-adaptation response to neurobehavioral toxicity induced by metal exposure in nematodes.

## Materials and Methods

### Reagents

The metal concentrations used in this study were referred to our previous description [17]–[18]. Three concentrations of HgCl_2_, and Pb(NO_3_)_2_ solutions were used in the current work, and they were 50 µM, 100 µM, and 200 µM, respectively. Metal concentrations of exposed solutions were analyzed by atomic absorption spectrophotometry (AAS; Pye-Unicam model SP9, Cambridge, UK). All the chemicals were obtained from Sigma-Aldrich (St. Louis, MO, USA).

### Strain preparation

Nematodes used in the present study were wild-type Bristol (N2), mutants of VC128 [*mtl-2(gk125)*], FX01770[*mtl-1(tm1770)*], and transgenic strains of *Ex*(P*mtl-1::GFP*), *Ex*(P*mtl-1::GFP*), *Ex*(P*mtl-1-mtl-1*), *Ex*(P*mtl-2-mtl-2*) and KC136 [*hsp-16.2::gfp*]. KC136 [*hsp-16.2::gfp*], an integrated transgenic line [16], is the gift from Dr. King L. Chow. They were maintained on nematode growth medium (NGM) plates seeded with *E. coli* OP50 at 20°C as described [44]. Gravid nematodes were washed off the plates into centrifuge tubes, and were lysed with a bleaching mixture (0.45 M NaOH, 2% HOCl). Age synchronous populations of L2- or L4-larval animals were obtained by the collection as described [45]. The collected nematodes were washed with double-distilled water, followed by washing with modified K medium once (50 mM NaCl, 30 mM KCl, 10 mM NaOAc, pH 5.5) [46]. Metal exposures were performed on L4-larval nematodes in 12-well sterile tissue culture plates, and all exposures were 12-h long and were carried out in 20°C incubator in the presence of food.

### Heat-shock experiments

Approximately 500 L4-stage larvae animals were heat stressed for 0.5, 1, 1.5, 2 h at 36°C, and approximately 500 L2-stage larvae animals were heat stressed for 1 h or 2 h at 36°C and afterwards further maintained at 20°C. All assays were replicated more than three times.

### Head thrash frequency

To assay the head thrashes, nematodes were washed with the double-distilled water, followed by washing with modified K medium. Every nematode was transferred into a microtiter well containing 60 µl of modified K medium on the top of agar. After a 1-min recovery period, the head thrashes were counted for 1-min. A thrash was defined as a change in the direction of bending at the mid body. Fifty nematodes were examined per treatment.

### Body bend frequency

To assay the body bends, nematodes were picked onto a second plate and scored for the number of body bends in an interval of 20 sec. A body bend was counted as a change in the direction of the part of the nematodes corresponding to the posterior bulb of the pharynx along the *y* axis, assuming that the nematode was traveling along the *x* axis. Fifty nematodes were examined per treatment.

### Analysis of KC136 transgenic strain

To analyze the changes of *hsp-16.2* expression patterns, the treated KC136 animals were allowed to settle for 10 min, and then pipetted onto an agar pad on a glass slide, mounted and observed for the fluorescent signals with a fluorescent microscope. Observations of the green fluorescent protein (GFP) at the pharyngeal bulb were recorded and color images were taken for the documentation of results with Magnafire® software (Olympus, Irving, TX, USA). The stress response was evaluated by the percentage of population with *gfp* expression. More than 50 nematodes were counted for the statistical analysis.

### DNA construct

The promoter fragments of *mtl-1* and *mtl-2* genes were selected according to the reference [35] and the suitable restriction enzyme sites for further sub-cloning into the pPD95_75 vector. The *mtl-1* (1197bp, PstI-XbaI) and *mtl-2* (551bp, XbaI/XhoI) promoter fragments were first sub-cloned into the pPD95_75 vector, and the *mtl-1* and *mtl-2* full length cDNA were further inserted into sites of BamHI/KpnI and XhoI/EcoRI of the pPD95_75 vector behind the fragments of P*mtl-1* and P*mtl-2*, respectively, to obtain the plasmids of P*mtl-1-mtl-1* and P*mtl-2-mtl-2*. The *mtl-1* and *mtl-2* full length cDNA were sub-cloned into the sites of BamHI/KpnI of the vector of pPD49_78 to obtain the plasmids of P*hsp-16-mtl-1* and P*hsp-16-mtl-2*. Transgenic worms were generated as previously described [47]. The plasmids were injected as a mix at 20 ng/µl using P*dop-1::rfp* as a transgenic marker, and the anticipated transgenic animals were identified with strong red signals [48]. The P*mtl-1* and P*mtl-2* plasmids were injected into the wild-type N2 nematodes to obtain the P*mtl-1::*GFP and P*mtl-2::*GFP transgenic animals.

To perform the rescue experiments, the constructs of P*mtl-1-mtl-1* and P*mtl-2-mtl-2* plasmids were injected into the *mtl-1* and *mtl-2* mutants, respectively. To perform the over-expression experiments, the constructs of P*hsp-16-mtl-1* and P*hsp-16-mtl-2* plasmids were injected into the wild-type N2 nematodes, respectively. Adult nematodes carrying P*hsp-16-mtl-1* and P*hsp-16-mtl-2* were placed on the NGM plates (0 hr), allowed to lay eggs for 4 hr at 20°C, and removed from the plates. The nematodes were heat shocked at 30°C for 4 hr, beginning at 40 hr (L2-larval stage), after the start of egg laying. The nematodes were cultured at 20°C again and subjecting to the locomotion behavior assay.

#### Reverse transcription-polymerase chain reaction

Total RNA was extracted using RNeasy Mini Kit (Qiagen). Total nematode RNA (∼1 µg) was reverse-transcribed using cDNA Synthesis kit (Bio-Rad Laboratories), and real-time PCR was performed using the primers for the target genes of *mtl-1* (forward primer, 5′-AGCTCAATTTGACTGCTGAA-3′; reverse primer, 5′-GAAACATTTTAATGAGCCGC-3′) and *mtl-2* (forward primer, 5′-CTGCCAGTGAGAAGAAATGC-3′; reverse primer, 5′-CGAACAATATCAATTAGTAGGAATTTG-3′), and reference gene of *ubp-1* (forward primer, 5′-CACTTGGTTCTTCGTCTTAG-3′; reverse primer, 5′-CCTCCTTGTCTTGAATCTTG-3′). Real-time PCR was run at the optimized annealing temperature of 58°C. The relative quantification of the *mtl-1* or *mtl-2* gene in comparison to the reference *ubq-1* gene was determined using the method described [49], and the final results were expressed as the relative expression ratio (between target gene and reference gene) in the treatments as compared to the ratio in the control.

### Statistical analysis

All data in this article were expressed as means ± SD. Graphs were generated using Microsoft Excel (Microsoft Corp., Redmond, WA). One-way analysis of variance (ANOVA) followed by a Dunnett's *t*-test was used to determine the significance of the differences between the groups. The probability levels of 0.05 and 0.01 were considered statistically significant.
